# Insights into *FGFR4* (rs351855 and rs7708357) Gene Variants, Ki-67 and p53 in Pituitary Adenoma Pathophysiology

**DOI:** 10.3390/ijms26157565

**Published:** 2025-08-05

**Authors:** Martyna Juskiene, Monika Duseikaite, Alvita Vilkeviciute, Egle Karinauske, Ieva Baikstiene, Jurgita Makstiene, Lina Poskiene, Arimantas Tamasauskas, Rasa Liutkeviciene, Rasa Verkauskiene, Birute Zilaitiene

**Affiliations:** 1 Institute of Endocrinology, Department of Endocrinology, Lithuanian University of Health Sciences, 50140 Kaunas, Lithuania; martyna.juskiene@lsmu.lt (M.J.); 2 Institute of Neuroscience, Lithuanian University of Health Sciences, Eiveniu Street 2, 50161 Kaunas, Lithuania; 3 Institute of Physiology and Pharmacology, Lithuanian University of Health Sciences, 44307 Kaunas, Lithuania; 4 Department of Pathology, Lithuanian University of Health Sciences, 44307 Kaunas, Lithuania

**Keywords:** pituitary adenoma, *FGFR4* (rs351855 and rs7708357), gene variants, ELISA, Ki-67, p53

## Abstract

To determine the association between *FGFR4* (rs351855 and rs7708357) gene variants, serum levels, and immunohistochemical markers (Ki-67 and p53) in pituitary adenoma (PA), a case-control study was conducted involving 300 subjects divided into two groups: the control group (*n* = 200) and a group of PA (*n* = 100). The genotyping of *FGFR4* rs351855 and rs7708357 was carried out using the real-time polymerase chain reaction (RT-PCR) method. The serum FGFR4 levels were measured using the ELISA method. Immunohistochemical analysis (Ki-67 and p53) was conducted. Statistical analysis of the data was performed using IBM SPSS Statistics 30.0 software. There were no statistically significant differences after analyzing the genotypes and alleles of *FGFR4* rs351855 and rs7708357 in patients with PA and control groups (all *p* > 0.05). After evaluating the distribution of genotypes and alleles of *FGFR4* rs351855 and rs7708357 in micro/macro, invasiveness, activity, and recurrence of PA and the control groups, the analysis showed no statistically significant differences between the groups (*p* > 0.05). Similarly, no significant differences in FGFR4 levels were observed between PA patients and control group (median (IQR): 3642.41 (1755.08) pg/mL vs. 3126.24 (1334.15) pg/mL, *p* = 0.121). Immunohistochemistry for Ki-67 revealed a labeling index (LI) of <1% in 25.5% of patients with PA, an LI of 1% in 10.9%, and an LI of >1% in 63.6% of patients. Further analyses showed no statistically significant associations with tumor size, invasiveness, activity, or recurrence. Immunohistochemistry for p53 revealed that macroadenomas had a significantly higher p53 H-score compared to microadenomas (median (IQR): 30.33 (28.68) vs. 18.34 (17.65), *p* = 0.005). Additionally, a moderate, statistically significant positive correlation between the Ki-67 LI and the p53 expression was found (Spearman’s ρ = 0.443, *p* = 0.003, *n* = 43). *FGFR4* variants and serum protein levels were not significantly associated with PA risk or tumor features. Conversely, immunohistochemical markers Ki-67 and p53 were more informative, with higher p53 expression in macroadenomas and a moderate positive correlation between Ki-67 and p53, highlighting their potential relevance in tumor growth assessment.

## 1. Introduction

Pituitary adenoma (PA) is typically a benign monoclonal neoplasm with an overall prevalence of approximately 16.7% in the general population [1]. However, most PAs are small and non-functional tumors, with only 0.16–0.2% classified as macroadenomas, defined by a diameter of ≥10 mm [1,2]. With modern imaging techniques and hormone testing, the detection rates of PA have increased. Autopsy studies suggest that these lesions may occur in up to 20% of the general population [1]. This increase is due to not only better detection of microadenomas but also macroadenomas [3]. Clinically, PAs are divided into non-functional pituitary adenomas (NFPAs) and functional pituitary adenomas (FPAs). Compared to FPAs, NFPAs are more challenging to diagnose and treat, as they typically remain asymptomatic until reaching a size sufficient to cause mass effect and compression symptoms [4]. Among the numerous clinical features of PA, invasiveness, which is defined as infiltration of adjacent structures such as the cavernous sinus, skull base bone, or sphenoid sinus, attracts significant clinical attention, as it complicates treatment and is associated with a variety of complications. Moreover, invasiveness has been shown to correlate with a poor prognosis [5].

The pathogenesis of PA is complex and not fully understood. It is generally believed to have a multifactorial etiology involving genetic factors, immunohistochemical markers, environmental factors, and other contributing elements. Recently, there has been growing interest in the identification of genetic markers that may influence the development and progression of PA.

Currently, 23 members of the fibroblast growth factor (FGF) ligand family have been identified, and their receptors are encoded by four independent genes, each producing multiple isoforms [6]. Several lines of evidence support the involvement of FGF/FGFR family members in pituitary tumorigenesis. Selected FGF ligands are overexpressed in pituitary tumors [7]. Additionally, the human endogenous FGF antisense gene (*GFG*) is expressed in the normal pituitary gland, where it restricts cell proliferation, whereas its expression is reduced in pituitary tumors [8]. Abbass et al. demonstrated altered expression of two FGFR family members in pituitary tumors [9]. Furthermore, Abbass along with colleagues, reported that FGFR4 undergoes NH2-terminal truncation, resulting in a pituitary tumor-derived FGFR4 variant (ptd-FGFR4) [9], initiated through alternative transcription initiation from a cryptic intronic promoter [10,11]. This oncogene receptor displaces N-cadherin from the cell membrane, disrupting normal cell adhesion [12]. In a large cohort of pituitary neoplasms, strong FGFR4 protein expression was observed more frequently in larger adenomas [13].

Beyond PA, FGFR4 has been extensively studied in other tumors, including breast, prostate, colorectal, and head and neck cancers. Its rs351855 variant has been linked to tumor progression, therapy resistance, and poor prognosis, while FGFR4 overexpression is considered a marker of aggressive behavior in several malignancies [14,15,16]. These findings highlight the broader oncological importance of FGFR4 and support the rationale for exploring its role in PA.

To predict the progression of PA, attention has been directed toward markers of invasiveness, such as the Ki-67 labeling index (LI) and p53. Ki-67 is a nuclear protein expressed at varying levels during the cell cycle. Its expression occurs during G1, S, and G2 phases, peaks at the beginning of mitosis and is absent during the quiescent phase of the cell cycle, G0. PAs with a Ki-67 LI of >3% have been associated with more aggressive tumor growth and worse prognosis [17]. p53, a transcription factor, plays an important role in DNA repair, cell cycle arrest, senescence, and apoptosis. It is noteworthy that p53 is inactivated in almost all tumors, and approximately 50% of these cases involve *TP53* mutations. Although the altered p53 expression is characteristic of many tumors, the p53 protein has rarely been studied in PA [18].

This study aims to investigate the effects of *FGFR4* gene variants in the promoter region (rs351855 and rs7708357), serum FGFR4 levels, and immunohistochemical markers (Ki-67 and p53) on the development and characteristics of PA.

## 2. Results

A case-control study was conducted involving 300 subjects divided into two groups: the control group (*n* = 200) and a group of PAs (*n* = 100). After forming the groups of subjects, an analysis of *FGFR4* rs351855 and rs7708357 was performed. The age median of PA patients was 51 years. The patients’ group was later divided into subgroups by the PA’s tumor size, hormonal activity, invasiveness, and recurrence. The age median of the control group was 53.5 years. The age and gender did not differ between study groups (*p* > 0.05). The demographic data of the subjects are presented in Table 1.

There were no statistically significant differences after analyzing the genotypes and alleles of *FGFR4* rs351855 and rs7708357 in patients with PA and control groups (all *p* > 0.05) (Table 2). In addition, binary logistic regression analysis did not show statistically significant differences between PA and the control group (Supplementary Material Table S1).

### 2.1. By Gender: Females and Males

No statistically significant differences were found in the genotypes and alleles of *FGFR4* rs351855 and rs7708357 when comparing male and female patients with PA to their respective control groups (all *p* > 0.05; Table 3 and Table 4).

Binary logistic regression analysis revealed no statistically significant differences between female or male PA and their corresponding controls (Supplementary Material Tables S2 and S3).

### 2.2. Associations of FGFR4 rs351855 and rs7708357 with Pituitary Adenoma’s Tumor Size

PA was divided into microadenomas and macroadenomas. After evaluating the distribution of genotypes and alleles of *FGFR4* rs351855 and rs7708357 in micro/macro PAs and the control groups, the analysis revealed no statistically significant differences between the groups (Table 5).

A binary logistic regression analysis was performed to evaluate the impact of *FGFR4* rs351855 and rs7708357 on micro/macro PA development. The analysis revealed no statistically significant differences between micro/macro PA and the control group (all *p* > 0.05) (Supplementary Material Table S4).

### 2.3. Associations of FGFR4 rs351855 and rs7708357 with Pituitary Adenoma’s Invasiveness

Analysis of adenoma invasiveness and the distribution of genotypes and alleles was performed by comparing the non-invasive PA group with the control group, as well as the invasive PA group with the control group. Evaluation of the genotype and allele distributions of *FGFR4* rs351855 and rs7708357 revealed no statistically significant differences between the groups (Table 6).

A binary logistic regression analysis between the non-invasive PA group and the control group, as well as between the invasive PA group and the control group of *FGFR4* rs351855 and rs7708357, did not show any statistically significant results (Supplementary Material Table S5).

### 2.4. Associations of FGFR4 rs351855 and rs7708357 with Pituitary Adenomas’ Activity

PA was also divided into active and non-active groups. After evaluating the distribution of genotypes and alleles of *FGFR4* rs351855 and rs7708357 in hormonal non-active/active PA and the control groups, the analysis revealed no statistically significant differences between the groups (Table 7).

Similarly to previous results, a binary logistic regression analysis between the non-active/active PA group and the control group of *FGFR4* rs351855 and rs7708357 did not show any statistically significant results (Supplementary Material Table S6).

### 2.5. Associations of FGFR4 rs351855 and rs7708357 with Pituitary Adenomas’ Recurrence

All patients with PA were also divided into PA without recurrence and PA with recurrence groups. After evaluating the distribution of genotypes and alleles of *FGFR4* rs351855 and rs7708357 in PA without recurrence and PA with recurrence, and the control groups, the analysis revealed no statistically significant differences between the groups (Table 8).

Binary logistic regression analysis comparing the PA groups without and with recurrence to the control group for *FGFR4* rs351855 and rs7708357 revealed no statistically significant associations. This finding is consistent with previous analyses, which also showed no significant associations (Table 9).

### 2.6. Serum FGFR4 Levels in Patients with PA and Controls

**Figure 1:**
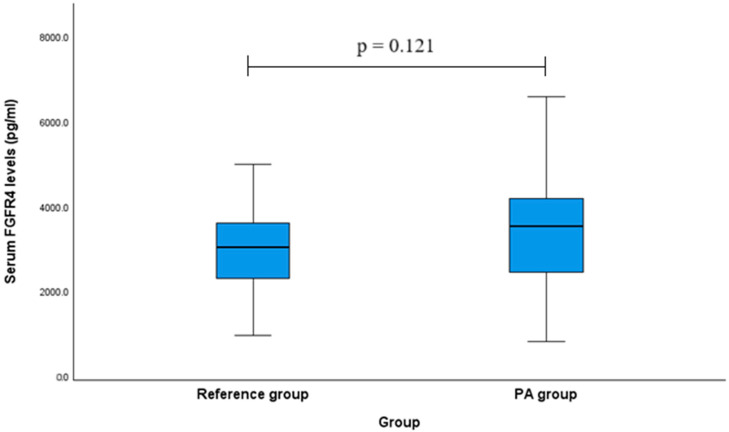
Serum FGFR4 levels in patients with PA and the reference group.

The study evaluated serum levels of FGFR4 in patients with PA compared to those in a reference group. However, no statistically significant differences were found between the serum FGFR4 levels in PA patients and the reference group. For FGFR4 levels, PA patients showed a median (IQR) of 3642.41 (1755.08) pg/mL compared to 3126.24 (1334.15) pg/mL in the reference group (*p* = 0.121; Figure 1).

Boxplots show the median, interquartile range (IQR), and full range (whiskers) of FGFR4 concentrations (pg/mL).

### 2.7. Ki-67 Labeling Index

In this part of the study, the 55 PA tissue samples were analyzed. The Ki-67 LI was evaluated in 36 females (65.5%) and 19 males (34.5%). The results revealed no statistically significant differences in the Ki-67 LI between females and males (*p* = 0.149).

Immunohistochemistry for Ki-67 revealed an LI of <1% in 25.5% of patients with PA, a Ki-67 LI of 1% in 10.9%, and a Ki-67 LI of >1% in 63.6% of patients. Further analyses revealed no statistical significance concerning tumor size (*p* = 0.333; Table 10), invasiveness (*p* = 0.666; Table 11), activity (*p* = 0.224; Table 12), or recurrence (*p* = 0.671; Table 13).

The analysis of the Ki-67 LI with the indicated genetic variations (*FGFR4* rs351855 and rs7708357) also revealed no statistically significant results, as shown in Table 14.

### 2.8. p53 Analysis in PA Tissues

Forty-five PA tissue samples were analyzed for p53. The p53 was evaluated in 28 women (62.2%) and 17 men (37.8%). The results revealed no statistically significant differences in the p53 H-score between women and men (*p* = 0.399). Immunohistochemistry for p53 revealed that macroadenomas had statistically significantly higher p53 H-score compared to in the microadenomas group (median (IQR): 30.33 (28.68) vs. 18.34 (17.65), *p* = 0.005). Further analyses revealed no statistical significance regarding the PA invasiveness, activity, or recurrence (Table 15).

**Figure 2:**
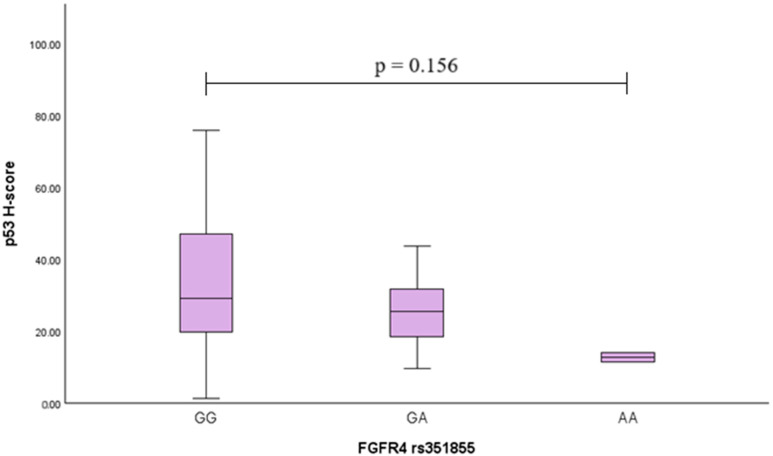
Association of *FGFR4* rs351855 genotype with the p53 H-score in PA patients. Boxplots illustrate the distribution of p53 H-scores across GG, GA, and AA genotypes. The median, interquartile range (IQR), and whiskers representing minimum and maximum values are shown.

**Figure 3:**
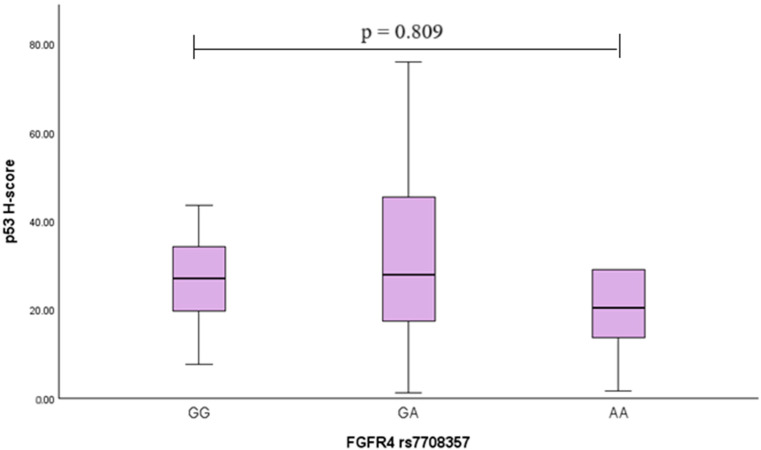
Associations of *FGFR4* rs7708357 genotype with the p53 H-score in PA patients. Boxplots illustrate the distribution of p53 H-scores across GG, GA, and AA genotypes. The median, interquartile range (IQR), and whiskers representing minimum and maximum values are shown.

To assess the association of the *FGFR4* rs351855 and rs7708357 variants with p53, the p53 H-score was calculated in different genotype groups, but no statistically significant differences were found (Figure 2 and Figure 3) (the Kruskal−Wallis test was used).

### 2.9. Correlation Between Ki-67 and p53

**Figure 4:**
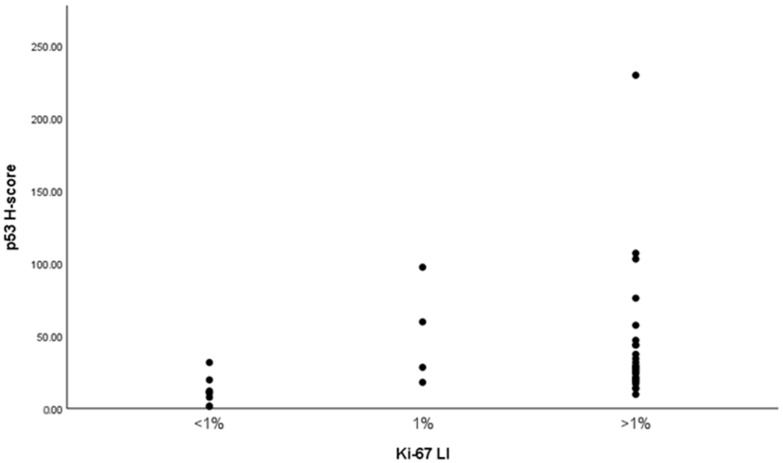
Correlation between the Ki-67 LI and the p53 H-score in PA. The scatter plot categorizes the Ki-67 LI into three groups: <1%, 1%, and >1%, with corresponding p53 H-scores displayed for each group. A nonparametric Spearman’s rank-order correlation demonstrated a moderate, statistically significant positive association between the Ki-67 LI and the p53 expression.

A nonparametric Spearman’s rank-order correlation was performed to assess the relationship between the Ki-67 LI and the p53 H-score (Figure 4). The results demonstrated a moderate, statistically significant positive correlation between the Ki-67 LI and p53 expression (Spearman’s ρ = 0.443, *p* = 0.003, *n* = 43). The 95% CI for the correlation coefficient ranged from 0.155 to 0.661. These findings suggest that increased proliferative activity, as measured by Ki-67, is associated with higher p53 expression in the studied samples. The scatter plot categorizes the Ki-67 LI into <1%, 1%, and >1%. It shows that higher Ki-67 levels are generally associated with higher p53 H-scores, particularly in the >1% group, where more variability and elevated H-scores are evident.

## 3. Discussion

It was found that the *FGFR4* gene variants analyzed in our study (rs351855 and rs7708357) do not play a prominent role in the development of PA. No statistically significant difference was found in serum FGFR4 levels, either.

Fibroblast growth factor receptors (FGFRs) are associated with various proliferative functions, and FGFR4 is differentially expressed in normal and neoplastic pituitary glands. Human pituitary tumors express a truncated FGFR4 isoform (ptd-FGFR4), whose transcription is initiated from an alternative downstream site. Analysis of the intronic sequences of *FGFR4* revealed a possible promoter within intron 4 (In4), including a classical TATA box with a possible transcription start site in intron 5 [11].

FGFR4 has been widely studied in other malignancies, such as breast, prostate, colorectal, gastric, and head and neck cancers, where its overexpression and polymorphic variants, particularly rs351855, have been linked to tumor progression, therapy resistance, and poor prognosis [14,15]. FGFR4 activation influences key oncogenic pathways such as the MAPK/ERK and PI3K/AKT signaling, which enhance cancer cell proliferation and survival. Recent studies have shown that FGFR4 inhibition can reverse therapy resistance, particularly in HER2-positive breast cancer, suggesting that FGFR4 is not only a prognostic marker but also a potential therapeutic target [16]. While our study did not find a significant association between *FGFR4* rs351855 or rs7708357 variants and PA characteristics, these results are consistent with the tissue-specific roles of *FGFR4* variants reported in other tumor types. Integrating data from broader oncological studies highlights that the lack of association in our cohort may reflect unique molecular mechanisms underlying pituitary adenomas, warranting further research with larger cohorts and functional analyses.

FGFR4 is widely overexpressed in human epithelial carcinomas [19,20,21], where it may contribute to tumor progression by various mechanisms [22,23,24,25]. About half of the population carries a homozygous or heterozygous *FGFR4*-G388R SNV, which has been associated with poor prognosis in various tumor types, such as adenocarcinomas of the breast, prostate, and colon, as well as squamous cell carcinomas and melanomas of the head and neck [22,24,26]. Studies by Gospodarowicz and Baird [27,28] have also shown that pituitary FGF plays an important role in the differentiation of pituitary cells and the paracrine regulation of hormone secretion, independent of its proliferative effect. These studies indicate that cultured lactotrophic and thyrotropic cells are more responsive to thyrotropin-releasing hormone after exposure to FGF, possibly due to stimulation of prolactin and thyroid-stimulating hormone synthesis [28].

Although the *FGFR4* was found to be an aggressive pituitary tumor marker [13,29,30], the study by Qian et al. [13] found that cytoplasmic expression of FGFR4 protein was observed in 57.8% of Japanese cases and 62.8% of Canadian cases, while FGFR4 reactivity was absent in all 10 normal adenohypophyseal tissues analyzed. FGFR4 expression in PA was mainly restricted to the cytoplasm, a pattern similar to that of rat pituitary cells transfected with human ptd-FGFR4 but different from that of cells transfected with wild-type FGFR4, which showed membrane localization of staining. The protein from primary human adenomas migrated as a 65 kDa species, which corresponds to the predicted size of ptd-FGFR4. FGFR4 protein expression was frequently found in adenomas containing GH, ACTH, or FSH/LH and was also found in null cell adenomas, but reactivity was relatively rare in prolactin-containing adenomas in both Japanese and Canadian groups. FGFR4 protein expression was more pronounced in macroadenomas than in microadenomas (*p* = 0.02), and high FGFR4 expression levels (moderate or greater density staining) were more frequently observed in macroadenomas than in microadenomas (*p* < 0.05) [13].

Ramirez et al., in their study, evaluated a large group of NFPA and found FGFR4 expression in most of the tumors, with variable levels of expression [31]. Histological analysis from another study group revealed that tumors with the prototypic *FGFR4* (G388) variant expressed higher levels of prolactin and lower levels of GH, whereas tumors with the polymorphic *FGFR4* (R388) variant showed increased GH expression relative to prolactin [32]. Tatento and colleagues, in an animal study, found that using a knock-in mouse model, FGFR4-R388 can promote the development of growth hormone pituitary tumors. In patients with acromegaly, the size of the pituitary tumor correlated with the hormone excess in the presence of *FGFR4*-R388 [30]. Ezzat et al. identified a new N-terminally truncated isoform of FGFR4 in human pituitary tumors, which they named ptd-FGFR4. This truncated receptor lacks a signal peptide and is localized in the cytoplasm, where it exhibits ligand-independent tyrosine phosphorylation. Ectopic expression of ptd-FGFR4 in NIH 3T3 cells leads to increased cell proliferation, anchorage-independent growth, and colony formation in vitro and induces tumorigenesis when injected in vivo [29].

Durcan and colleagues investigated the relationship between FGFR4 expression and radiological, pathological, and clinical parameters in PA. They found that median H-scores for FGFR4 were higher in patients without remission, in those with residual lesions, and in T2-hyperintense adenomas (*p* < 0.05). Adenomas with Ki-67 expression of ≥3% had higher FGFR4 expression levels than those with <3% expression (*p* = 0.002). There was a weak positive correlation between the H-score and Ki-67 (*p* = 0.011; r = 0.201), and the authors concluded that higher levels of FGFR4 in PA could be used as a marker for more aggressive tumor behavior [33].

Functional variants in the *FGFR4* gene, specifically the R388 variant, have been linked to silent macrocorticotropinomas, whereas the G388 variant is more commonly found in small, hormonally active tumors [30]. These results indicate that the transmembrane *FGFR4* variants can modulate cell growth and sensitivity to glucocorticoid hormone negative feedback through various STAT3 modifications relevant to human forms of Cushing’s syndrome [30].

Another study analyzed 76 patients who underwent the first transsphenoidal surgery. All patients were genotyped for the G388 variant. FGFR4 expression was assessed by real-time PCR in 18 corticotrophinomas. Homozygosis for the *FGFR4* glycine (Gly388) allele was associated with reduced disease-free survival in the univariate analysis (*p* = 0.028). Male gender (*p* = 0.036), lack of pathology confirmation (*p* = 0.009), and cortisol levels more than 2 μg/dl in the early postoperative period (*p* < 0.001) were also significant predictors of Cushing’s disease recurrence. FGFR4 overexpression was found in 44% of corticotrophinomas and was associated with a lower postoperative remission rate (*p* = 0.009). These data suggest that homozygosity for the *FGFR4* Gly388 allele and FGFR4 overexpression are associated with a higher frequency of postoperative recurrence and persistence of Cushing’s disease [34].

Ki-67 is considered a biomarker of aggressive tumor behavior in the World Health Organization (WHO) classification of PA. Increased proliferation rates are associated with more aggressive behavior, while low proliferation rates are typically observed in non-invasive tumors [35,36]. Some studies have examined the combined expression of p53 and Ki-67 with tumor invasiveness, aggressiveness, and recurrence [37]. Honegger et al. concluded that Ki-67 expression correlates positively with the growth velocity of PA, while invasive behavior is independent of the Ki-67 [38]. Chang et al. identified cavernous sinus invasion as a stronger predictor of recurrence and recommended caution in postoperative radiotherapy [39]. In a multivariate analysis, gender and parasellar tumor extension were the best predictors of persistent disease [40].

In 1991, Kitz et al. reported a significantly higher Ki-67 LI in invasive than in non-invasive adenomas [41]. Mastronardi et al. reported that the Ki-67 LI was higher in functioning than in nonfunctioning tumors, particularly in ACTH adenomas [42]. Thapar et al. reported that hormone-secreting adenomas had a significantly higher mean Ki-67 LI (3.25%) than non-functioning adenomas (2.06%). Other case series focusing on prolactinomas have demonstrated that a higher Ki-67 LI correlates with higher prolactin levels and larger macroprolactinomas [43]. In 2010, Pawlikowski et al. suggested that plurihormonal adenomas, especially ACTH-secreting tumors, have higher Ki-67 LIs compared to monohormonal tumors. In our study, we also revealed that there is a statistically significant positive correlation between the Ki-67 LI and the p53 expression. A high Ki-67 LI in ACTH-secreting adenomas was reported in another study as well. For gonadotropin-secreting and null cell adenomas, other studies showed low Ki-67 proliferation marker levels. A study conducted on a relatively low number of pituitary carcinoma samples concluded that the mean Ki-67 LI was 2.6% for primary tumors and 11% for metastatic tumors. About 40% of APT/PC in this survey had Ki-67 levels of ≥10%, compared with 3% of 374 tumors in the Lyon surgical series [44].

*TP53* is a tumor suppressor gene and one of the most frequently mutated genes in cancer. Mutations are associated with high nuclear expression of the encoded p53 protein due to reduced protein degradation, but can also lead to a complete absence of the protein. In a multicenter study of 701 PAs, p53 staining in more than 10% of tumor cells was observed in a subgroup of 36 cases classified as atypical according to the WHO 2004 classification [45]. However, the term “atypical adenoma”—previously defined by a Ki-67 LI of >3%, increased mitotic activity, and strong p53 immunoreactivity—was removed in the 2017 WHO classification due to limited prognostic utility. According to the 2022 WHO guidelines, p53 immunostaining may still be assessed in selected cases to provide additional clinical information [46,47].

Numerous studies have demonstrated that immunohistochemistry results for p53 and Ki-67 have biological and clinical relevance, particularly in relation to tumor proliferation, aggressiveness, and recurrence. Both Ki-67 and p53 are already used in clinical practice as prognostic markers, as recommended by the WHO and the European Society of Endocrinology (ESE). In cases where the Ki-67 LI and the p53 expression are elevated, patients are advised to undergo more intensive clinical and radiological surveillance. Additionally, in the presence of residual tumor or a high risk of recurrence, adjunctive radiotherapy is often considered. Considering the strong role of p53 and Ki-67 in the pathogenesis of PA, the development of novel targeted therapeutic agents aimed at these markers could potentially improve treatment efficacy and patient outcomes.

Although our study did not identify statistically significant associations between *FGFR4* variants and PA, these null results are informative as they suggest that these *FGFR4* variants may have a limited role in PA pathogenesis, helping to refine current genetic models of this tumor type. Our findings also complement and challenge previous reports by confirming that genetic variation in *FGFR4* does not uniformly contribute to tumor behavior across different tumor types, underscoring the tissue-specific nature of FGFR4 involvement. By integrating genetic, protein-level, and immunohistochemical data, this study provides a broader context in which markers such as Ki-67 and p53 emerge as more reliable indicators of PA aggressiveness, even in the absence of significant genetic associations. Future research with larger, multicenter cohorts will be essential to confirm these findings and to detect potential smaller genetic effects that may not have been captured due to the current sample size.

## 4. Materials and Methods

### 4.1. Study Design

The Kaunas Regional Biomedical Research Ethics Committee granted permission (No. BE-2-47, issued on 25 December 2016) for the case-control study, which was conducted in the Laboratory of Ophthalmology and Department of Neurosurgery at Lithuanian Health Sciences University Hospital. All participants were provided with a comprehensive explanation of the study’s structure and objectives. Informed consent was obtained from each participant under ethical research standards.

### 4.2. Study Population

Study participants comprised 100 patients with a diagnosis of PA, and the control group involved 200 subjects. The control group was created by taking into consideration the distribution of age and gender in the PA group. Therefore, the medians of the patients’ age of the control group and the PA group did not differ significantly (*p* < 0.05). Using the global PA prevalence (20%) and the minor allele frequencies of rs351855 (A = 30.1%) and rs7708357 (A = 40.4%) from the dbSNP database [48], we calculated that our sample sizes (100 PA cases and 200 controls) provide less than 80% statistical power, suggesting that future studies should include larger cohorts to achieve sufficient power.

Patients with PA were selected based on the following inclusion criteria:

(1) Determined and confirmed PA via MRI or CT and/or histopathological examination were included;

(2) The patient’s general good condition;

(3) Patient’s consent to take part in the study;

(4) Age of ≥18 years,

(5) no other brain or other localized tumors.

Patients with PAs’ exclusion criteria were as follows:

(1) Patients without a confirmed diagnosis of PA through imaging (MRI/CT) and/or histopathological examination were excluded.

(2) Patients younger than 18 years of age, those with significant health issues that could impact their participation, or the study results were excluded.

(3) Patients in poor overall health, as determined by their clinical assessment, were excluded.

(4) Patients with other brain tumors, tumors in other locations, intracranial infections, demyelinating lesions, or cerebrovascular diseases were excluded.

(5) Patients who did not provide informed consent were excluded from the study.

The control group inclusion criteria were as follows:

(1) Participants were required to be in good overall health, with no history of PAs or other significant medical conditions that could influence study outcomes.

(2) Only individuals aged 18 years or older were included to ensure comparability with the patient cohort.

(3) Participants must have no history of brain tumors, extracranial tumors, intracranial infections, demyelinating diseases, cerebrovascular disorders, or other major systemic illnesses.

(4) Individuals with any prior diagnosis or clinical/imaging evidence of pituitary disorder.

(5) All participants provided written informed consent.

The control group exclusion criteria included the following:

(1) Individuals with significant health conditions, including pituitary disorders, brain tumors, or major systemic diseases, were excluded.

(2) Individuals under 18 years of age were excluded.

(3) Participants with a history of pituitary or brain disorders were not included.

(4) Individuals who did not provide informed consent were excluded from the study.

### 4.3. DNA Extraction and Genotyping

The genotyping of *FGFR4* (rs351855 and rs7708357) was performed at the Laboratory of Ophthalmology, Neuroscience Institute, Lithuanian University of Health Sciences (LUHS).

DNA was extracted from 200 μL venous blood (white blood cells) using a DNA purification kit based on the magnetic beads method (MagJET Genomic DNA Kit, Thermo Scientific, Waltham, MA, USA) or the silica-based membrane technology utilizing a genomic DNA extraction kit (GeneJET Genomic DNA Purification Kit, Thermo Scientific, Waltham, MA, USA), according to the manufacturer’s recommendations. The quality and concentration of the extracted DNA were evaluated using a Cary 60 UV–Vis spectrophotometer (Agilent Technologies, Penang, Malaysia). Only samples exhibiting an A260/A280 ratio within the range of 1.8 to 2.0 were selected for genotyping.

Single-nucleotide variants of *FGFR4* (rs351855 and rs7708357) were carried out using the real-time polymerase chain reaction (RT-PCR) method. TaqMan^®^ Genotyping Assays were used to identify SNVs following the manufacturer’s instructions, using the StepOne Plus system (Applied Biosystems, Waltham, MA, USA). To verify accuracy, 5% of the samples were reanalyzed for two SNVs, demonstrating consistent results between the initial and repeat genotyping. Each PCR reaction was carried out in a final volume of 10 µL, consisting of 5 µL TaqMan Genotyping Master Mix, 0.5 µL TaqMan SNP Genotyping Assay Mix, 3.5 µL nuclease-free water, and 1 µL of genomic DNA (20 ng/µL).

The specific TaqMan^®^ SNV Genotyping Assays used for each SNV are detailed below:

rs351855 C___3166614_10;

Sequence [VIC/FAM]: CTTGGCTGTGCTCCTGCTGCTGGCC[A/G]GGCTGTATCGAGGGCAGGCGCTCCA

rs7708357 C__11270571_20;

Sequence [VIC/FAM]:

TTGCATTGCTACCCAGATGCTGCTG[A/G]TCTGGGGAAGGAGTGGGGGTCACAC.

### 4.4. Serum Level’s Measurement

Peripheral venous blood was collected and allowed to clot at room temperature for 30 min. Samples were then centrifuged, and the serum was carefully separated from the cellular fraction and transferred into 2 mL tubes and stored at −80 °C until analysis. Serum FGFR4 levels were measured in duplicate in control subjects and patients with PA. The determination was performed by enzyme-linked immunosorbent assay (ELISA) using the antibody Of human fibroblast growth factor receptor 4 (FGFR4) ELISA kit (Cat. No. Abx252459; standard curve sensibility range: 78.13–5000 pg/mL, sensitivity: 46.9 pg/mL). Serum levels were analyzed according to the manufacturer’s instructions using a Multiskan FC Microplate Photometer (Thermo Scientific, Waltham, MA, USA) at 450 nm.

### 4.5. Evaluation of Ki-67 and p53

The evaluation of the Ki-67 LI and the p53 expression was performed at the Department of Pathological Anatomy, LUHS, by a qualified pathologist. Immunohistochemical reactions of the Ki-67 LI and p53 protein biomarkers were carried out using the automated Ventana BenchMark ULTRA PLUS staining system (Roche Diagnostics, Basel, Switzerland), following the manufacturer’s recommendations.

Immunohistochemical staining was detected using monoclonal antibodies: Ki-67 (clone SP6, Vitro S.A., Sevilla, Spain) and p53 (clone DO-7, Roche Diagnostics, Basel, Switzerland). After performing Ki-67 and p53 immunohistochemical reactions, the images were digitized with a Pannoramic 250 FLASH III scanner (3DHISTECH Ltd., Budapest, Hungary). The evaluation of the digitized images for Ki-67 and p53 was conducted using the 3DHISTECH SlideViewer 2.9.0. software (3DHISTECH Ltd., Budapest, Hungary), based on the WHO Classification of Endocrine and Neuroendocrine Tumors [48].

For both biomarkers, the most positively stained regions (hotspots) were selected for assessment. A total of 300 tumour cell nuclei were evaluated fore each sample, and the percentage of positively stained nuclei was calculated to determine the Ki-67 LI. For the evaluation of p53, the tumour cell nuclei were divided into the categories “Negative (−)”, “Weakly positive (+)”, “Moderately positive (++)”, or “Strongly positive (+++)”, taking into account the color of the nuclear staining, its intensity, and its distribution throughout the nucleus. Morphologically unclear nuclei were excluded from the analysis.

To provide an objective quantitative measure of p53 staining intensity, the H-score was calculated using the formula:

H-score = (1 × % weakly stained nuclei) + (2 × % moderately stained nuclei) + (3 × % strongly stained nuclei).

This scoring system allowed for a semi-quantitative evaluation of p53 expression.

### 4.6. Statistical Analysis

Statistical analysis was performed using the SPSS/W 30.0 software (Statistical Package for the Social Sciences for Windows, Inc., Chicago, IL, USA). Descriptive data are reported as absolute frequencies with percentages, and continuous variables are expressed as medians with interquartile ranges (IQRs). The Hardy–Weinberg equilibrium (HWE) for the *FGFR4* single-nucleotide variants (SNVs) rs351855 and rs7708357 was assessed using the chi-square (χ^2^) test to compare observed and expected genotype frequencies. Genotypic and allelic distributions between PA patients and control subjects were compared using the chi-square test. Binomial logistic regression was used to evaluate the association between *FGFR4* genotypes and the risk of PA development, with results expressed as odds ratios (ORs) with corresponding 95% confidence intervals (CIs). The selection of the best genetic model was based on the Akaike Information Criterion (AIC); therefore, the best genetic models were those with the lowest AIC values. For immunohistochemical markers, nonparametric analyses were performed. The Mann–Whitney U test was used to compare p53 H-scores between PA subgroups. Correlation between the Ki-67 labeling index (LI) and the p53 H-score was assessed using Spearman’s rank-order correlation coefficient (ρ). No multiple comparison correction was applied, as the analyses were limited to predefined SNVs and markers based on specific hypotheses. Differences were considered statistically significant when *p* < 0.05.

## 5. Conclusions

Our study found no significant association between *FGFR4* variants or serum FGFR4 levels and PA pathogenesis or clinical behavior. In contrast, immunohistochemical markers Ki-67 and p53 demonstrated greater relevance, with macroadenomas showing significantly higher p53 expression and a moderate positive correlation between Ki-67 and p53. These findings emphasize the potential utility of Ki-67 and p53 in characterizing tumor growth, while the genetic variants studied appear to play a limited role. Further studies with larger cohorts and functional analyses are needed to confirm these observations and explore underlying molecular mechanisms.

This study is among the few to simultaneously evaluate *FGFR4* variants (rs351855 and rs7708357), serum FGFR4 levels, and immunohistochemical markers (Ki-67 and p53) in PA. The integration of genetic, protein-level, and histological data provides a more comprehensive understanding of the potential role of FGFR4 in PA pathogenesis. Unlike many prior studies, our work investigates both genetic variants and functional markers within the same patient cohort, offering valuable insights even in the absence of statistically significant associations.

However, several limitations should be noted. First, the relatively small sample size limits the statistical power, particularly for detecting small effect sizes of genetic variants. Second, this was a single-center study, which may limit the generalizability of the findings to broader populations. Third, the lack of functional assays or pathway analyses restricts our ability to infer the biological mechanisms underlying the observed correlations. Future studies with larger, multicenter cohorts and additional mechanistic investigations are needed to confirm and expand on these findings.

## Figures and Tables

**Table 1:** Demographic characteristics of study subjects.

Characteristics	Group		*p*-Value
	PA, *n* (%) (*n* = 100)	Control, *n* (%) (*n* = 200)	
Age median (IQR)	51 (21)	53.5 (40)	0.655 *
Gender, *n* (%)			0.294 **
Females	64 (64)	140 (70)	
Males	36 (36)	60 (30)	
Tumor size, *n* (%)		-	-
Micro PA	38 (38)		
Macro PA	62 (62)		
Hormonal activity, *n* (%)		-	-
Active	59 (59)		
Non-active	41 (41)		
Invasiveness, *n* (%)		-	-
Invasive	53 (53)		
Non-invasive	47 (47)		
Recurrence, *n* (%)		-	-
PA without recurrence	77 (77)		
PA with recurrence	23 (23)		

* Mann−Whitney U test; ** Pearson chi-square test.

**Table 2:** Distributions of *FGFR4* rs351855 and rs7708357 genotypes and alleles in patients with PA and control groups.

Gene	Genotype/Allele	PA Group *n* (%) (*n* = 100)	Control Group *n* (%) (*n* = 200)	*p*-Value	*p*-Value HWE
*FGFR4* (rs351855)	GG	45 (45)	95 (47.5)	0.885	0.134
	GA	49 (49)	92 (46)		
	AA	6 (6)	13 (6.5)		
	In total:	100 (100)	200 (100)		
	Allele:			0.800	
	G	139 (69.5)	282 (70.5)		
	A	61 (30.5)	118 (29.5)		
*FGFR4* (rs7708357)	GG	40 (40)	86 (43)	0.755	0.791
	GA	49 (49)	89 (44.5)		
	AA	11 (11)	25 (12.5)		
	In total:	100 (100)	200 (100)		
	Allele:			0.855	
	G	129 (64.5)	261 (65.3)		
	A	71 (35.5)	139 (34.7)		

**Table 3:** Distributions of *FGFR4* rs351855 and rs7708357 genotypes and alleles in female patients with PA and the control group.

Gene	Genotype/Allele	PA Group Females (*n* = 64) *n* (%)	Control Group Females (*n* = 140) *n* (%)	*p*-Value
*FGFR4* (rs351855)	GG	27 (42.2)	69 (49.3)	0.490
	GA	34 (53.1)	62 (44.3)	
	AA	3 (4.7)	9 (6.4)	
	In total:	64 (100)	140 (100)	
	Allele:			0.581
	G	88 (68.75)	200 (71.4)	
	A	40 (31.25)	80 (28.6)	
*FGFR4* (rs7708357)	GG	25 (39.1)	56 (40)	0.726
	GA	33 (51.6)	66 (47.1)	
	AA	6 (9.4)	18 (12.9)	
	In total:	64 (100)	140 (100)	
	Allele:			0.803
	G	83 (64.8)	178 (63.6)	
	A	45 (35.2)	102 (36.4)	

**Table 4:** Distributions of *FGFR4* rs351855 and rs7708357 genotypes and alleles in male patients with PA and the control group.

Gene	Genotype/Allele	PA Group Males (*n* = 36) *n* (%)	Control Group Males (*n* = 60) *n* (%)	*p*-Value
*FGFR4* (rs351855)	GG	18 (50)	26 (43.3)	0.727
	GA	15 (41.7)	30 (50)	
	AA	3 (8.3)	4 (6.7)	
	In total:	36 (100)	60 (100)	
	Allele:			0.716
	G	51 (70.8)	82 (68.3)	
	A	21 (29.2)	38 (31.7)	
*FGFR4* (rs7708357)	GG	15 (41.7)	30 (50)	0.730
	GA	16 (44.4)	23 (38.3)	
	AA	5 (13.9)	7 (11.7)	
	In total:	36 (100)	60 (100)	
	Allele:			0.450
	G	46 (63.9)	83 (69.2)	
	A	26 (36.1)	37 (30.8)	

**Table 5:** Distributions of *FGFR4* rs351855 and rs7708357 genotypes and alleles in PA and control groups by PA tumor size.

Gene	Genotype/ Allele	Control Group (*n* = 200) *n* (%)	Micro PA (*n* = 38) *n* (%)	*p*-Value	Macro PA (*n* = 62) *n* (%)	*p*-Value
*FGFR4* (rs351855)	GG	95 (47.5)	15 (39.5)	0.577	30 (48.4)	0.992
	GA	92 (46)	21 (55.3)		28 (45.2)	
	AA	13 (6.5)	2 (5.3)		4 (6.5)	
	In total:	200 (100)	38 (100)		62 (100)	
	Allele:			0.554		0.920
	G	282 (70.5)	51 (67.1)		88 (71)	
	A	118 (29.5)	25 (32.9)		36 (29)	
*FGFR4* (rs7708357)	GG	86 (43)	15 (39.5)	0.836	25 (40.3)	0.494
	GA	89 (44.5)	17 (44.7)		32 (51.6)	
	AA	25 (12.5)	6 (15.8)		5 (8.1)	
	In total:	200 (100)	38 (100)		62 (100)	
	Allele:			0.568		0.857
	G	261 (65.3)	47 (61.8)		82 (66.1)	
	A	139 (34.7)	29 (38.2)		42 (33.9)	

**Table 6:** Distributions of *FGFR4* rs351855 and rs7708357 genotypes and alleles in PA and control groups by PA invasiveness.

Gene	Genotype/ Allele	Control Group (*n* = 200) *n* (%)	Non-Invasive PA (*n* = 47) *n* (%)	*p*-Value	Invasive PA (*n* = 53) *n* (%)	*p*-Value
*FGFR4* (rs351855)	GG	95 (47.5)	17 (36.2)	0.348	28 (52.8)	0.787
	GA	92 (46)	27 (57.4)		22 (41.5)	
	AA	13 (6.5)	3 (6.4)		3 (5.7)	
	In total:	200 (100)	47 (100)		53 (100)	
	Allele:			0.288		0.533
	G	282 (70.5)	61 (64.9)		78 (73.6)	
	A	118 (29.5)	33 (35.1)		28 (26.4)	
*FGFR4* (rs7708357)	GG	86 (43)	15 (31.9)	0.353	25 (47.2)	0.367
	GA	89 (44.5)	24 (51.1)		25 (47.2)	
	AA	25 (12.5)	8 (17)		3 (5.7)	
	In total:	200 (100)	47 (100)		53 (100)	
	Allele:			0.156		0.286
	G	261 (65.3)	54 (57.4)		75 (70.8)	
	A	139 (34.7)	40 (42.6)		31 (29.2)	

**Table 7:** Distributions of *FGFR4* rs351855 and rs7708357 genotypes and alleles in patients with PA and control groups by PA activity.

Gene	Genotype/ Allele	Control Group (*n* = 200) *n* (%)	Non-Active PA (*n* = 41) *n* (%)	*p*-Value	Active PA (*n* = 59) *n* (%)	*p*-Value
*FGFR4* (rs351855)	GG	95 (47.5)	20 (48.8)	0.713	25 (42.4)	0.435
	GA	92 (46)	17 (41.5)		32 (54.2)	
	AA	13 (6.5)	4 (9.8)		2 (3.4)	
	In total:	200 (100)	41 (100)		59 (100)	
	Allele:			0.858		0.833
	G	282 (70.5)	57 (69.5)		82 (69.5)	
	A	118 (29.5)	25 (30.5)		36 (30.5)	
*FGFR4* (rs7708357)	GG	86 (43)	13 (31.7)	0.360	27 (45.8)	0.866
	GA	89 (44.5)	23 (56.1)		26 (44.1)	
	AA	25 (12.5)	5 (12.2)		6 (10.2)	
	In total:	200 (100)	41 (100)		59 (100)	
	Allele:			0.344		0.608
	G	261 (65.3)	49 (59.8)		80 (67.8)	
	A	139 (34.7)	33 (40.2)		38 (32.2)	

**Table 8:** Distributions of *FGFR4* rs351855 and rs7708357 genotypes and alleles in patients with PA and control groups by PA recurrence.

Gene	Genotype/ Allele	Control Group (*n* = 200) *n* (%)	PA Without Recurrence (*n* = 77) *n* (%)	*p*-Value	PA with Recurrence (*n* = 23) *n* (%)	*p*-Value
*FGFR4* (rs351855)	GG	95 (47.5)	34 (44.2)	0.316	11 (47.8)	0.150
	GA	92 (46)	41 (53.2)		8 (34.8)	
	AA	13 (6.5)	2 (2.6)		4 (17.4)	
	In total:	200 (100)	77 (100)		23 (100)	
	Allele:			0.948		0.459
	G	282 (70.5)	109 (70.8)		30 (65.2)	
	A	118 (29.5)	45 (29.2)		16 (34.8)	
*FGFR4* (rs7708357)	GG	86 (43)	27 (35.1)	0.465	13 (56.5)	0.339
	GA	89 (44.5)	40 (51.9)		9 (39.1)	
	AA	25 (12.5)	10 (13)		1 (4.3)	
	In total:	200 (100)	77 (100)		23 (100)	
	Allele:			0.354		0.140
	G	261 (65.3)	94 (61)		35 (76.1)	
	A	139 (34.7)	60 (39)		11 (23.9)	

**Table 9:** Binary logistic regression analysis of *FGFR4* rs351855 and rs7708357 in the PA and control groups by PA recurrence.

*FGFR4* (rs351855)					
Model		Genotype/Allele	OR (95% CI)	*p*-value	AIC
PA without recurrence					
Codominant		GA vs. GG	1.245 (0.727–2.131)	0.424	328.894
		AA vs. GG	0.430 (0.092–2.004)	0.282	
Dominant		GA + AA vs. GG	1.144 (0.675–1.941)	0.617	329.182
Recessive		AA vs. GG + GA	0.384 (0.085–1.741)	0.214	327.535
Overdominant		GA vs. GG + AA	1.337 (0.789–2.265)	0.280	328.263
Additive		A	0.984 (0.631–1.535)	0.944	329.428
PA with recurrence					
Model		Genotype/Allele	OR (95% CI)	*p*-value	AIC
Codominant		GA vs. GG	0.751 (0.289–1.951)	0.557	148.963
		AA vs. GG	2.657 (0.737–9.584)	0.135	
Dominant		GA + AA vs. GG	0.987 (0.416–2.342)	0.976	150.038
Recessive		AA vs. GG + GA	3.028 (0.898–10.216)	0.074	147.312
Overdominant		GA vs. GG + AA	0.626 (0.254–1.543)	0.309	148.969
Additive		A	1.298 (0.665–2.537)	0.445	149.464
*FGFR4* (rs7708357)					
Model		Genotype/Allele	OR (95% CI)	*p*-value	AIC
PA without recurrence					
Codominant		GA vs. GG	1.432 (0.809–2.534)	0.218	329.890
		AA vs. GG	1.274 (0.544–2.985)	0.577	
Dominant		GA + AA vs. GG	1.397 (0.810–2.410)	0.230	327.967
Recessive		AA vs. GG + GA	1.045 (0.476–2.292)	0.913	329.421
Overdominant		GA vs. GG + AA	1.348 (0.796–2.284)	0.266	328.195
Additive		A	1.201 (0.816–1.769)	0.353	328.571
PA with recurrence					
Model	Genotype/Allele		OR (95% CI)	*p*-value	AIC
Codominant		GA vs. GG	0.669 (0.272–1.646)	0.381	149.601
		AA vs. GG	0.265 (0.033–2.123)	0.211	
Dominant		GA + AA vs. GG	0.580 (0.243–1.386)	0.221	148.522
Recessive		AA vs. GG + GA	0.318 (0.041–2.465)	0.273	148.379
Overdominant		GA vs. GG + AA	0.802 (0.332–1.938)	0.624	149.795
Additive		A	0.593 (0.292–1.204)	0.148	147.781

OR—odds ratio; CI—confidence interval; AIC—Akaike Information Criteria; *p*-value—significance level (statistically significant when *p* < 0.05).

**Table 10:** Ki-67 labeling index considering the size of PA.

Ki-67 LI	Tumor Size		*p*-Value
	Micro PA (*n* = 20) (%)	Macro PA (*n* = 35) (%)	
<1%	7 (35)	7 (20)	0.333
1%	1 (5)	5 (14.3)	
>1%	12 (60)	23 (65.7)	

**Table 11:** Ki-67 labeling index considering the invasiveness of PA.

Ki-67 LI	Invasiveness		*p*-Value
	Non-Invasive PA (*n* = 22) (%)	Invasive PA) (*n* = 33) (%)	
<1%	7 (31.8)	7 (21.2)	0.666
1%	2 (9.1)	4 (12.1)	
>1%	13 (59.1)	22 (66.7)	

**Table 12:** Ki-67 labeling index considering the activity of PA.

Ki-67 LI	Activeness		*p*-Value
	Non-Active PA (*n* = 24) (%)	Active PA (*n* = 31) (%)	
<1%	5 (20.8)	9 (29)	0.224
1%	1 (4.2)	5 (16.1)	
>1%	18 (75)	17 (54.8)	

**Table 13:** Ki-67 labeling index considering the recurrence of PA.

Ki-67 LI	Recurrence		*p*-Value
	PA Without Recurrence (*n* = 37) (%)	PA with Recurrence (*n* = 18) (%)	
<1%	9 (24.3)	5 (27.8)	0.671
1%	5 (13.5)	1 (5.6)	
>1%	23 (62.2)	12 (66.7)	

**Table 14:** Ki-67 labeling index associations with *FGFR4* rs351855 and rs7708357.

Gene, SNV	Genotype/Allele	Ki-67 LI			*p*-Value
		<1%	1%	>1%	
*FGFR4* rs351855	GG	7 (50)	4 (66.7)	14 (40)	0.754
	GA	6 (42.9)	2 (33.3)	19 (54.3)	
	AA	1 (7.1)	0 (0)	2 (5.7)	
	In total:	14 (100)	6 (100)	35 (100)	
	Allele:				0.518
	G	20 (71.4)	10 (83.3)	47 (67.1)	
	A	8 (28.6)	2 (16.7)	23 (32.9)	
*FGFR4* rs7708357	GG	8 (57.1)	2 (33.3)	16 (45.7)	0.885
	GA	5 (35.7)	3 (50)	15 (42.9)	
	AA	1 (7.1)	1 (16.7)	4 (11.4)	
	In total:	14 (100)	6 (100)	35 (100)	
	Allele:				0.556
	G	21 (75)	7 (58.3)	47 (67.1)	
	A	7 (25)	5 (41.7)	23 (32.9)	

**Table 15:** Associations of clinical features of PA with the p53 H score.

PA Subgroups	p53 H Score Median (IQR)	*p*-Value *
Micro PA	18.34 (17.65)	**0.005**
Macro PA	30.33 (28.68)	
Non-invasive PA	21.32 (17.65)	0.324
Invasive PA	27.5 (26.25)	
Non-active PA	21.02 (17.65)	0.068
Active PA	28.33 (49.5)	
PA without recurrence	28.66 (25.41)	0.360
PA with recurrence	21.02 (14.65)	

* Mann−Whitney U test was used; PA—pituitary adenoma.

## Data Availability

The datasets used and/or analyzed during the current study are available from the corresponding author on reasonable request.

## Supplementary Materials

The following supporting information can be downloaded at: https://www.mdpi.com/article/10.3390/ijms26157565/s1.

## Abbreviations

ACTH	Adrenocorticotropic Hormone
APT/PC	Atypical Pituitary Tumor/Pituitary Carcinom
CT	Computed Tomography
DNA	Deoxyribonucleic Acid
ELISA	Enzyme-Linked Immunosorbent Assay
FGF	fibroblast growth factor
FPA	functional pituitary adenoma
FS H/LH	Follicle-Stimulating Hormone/Luteinizing Hormone
GH	Growth Hormone
GFG	Human Endogenous FGF Antisense Gene
MRI	magnetic resonance imaging
NFPA	non-functional pituitary adenomas
PA	pituitary adenoma
RT-PCR	real-time polymerase chain reaction
SNV	single-nucleotide variant
STAT3	Signal Transducer and Activator of Transcription 3
