# Supplementary material for: Insights into FGFR4 (rs351855 and rs7708357) Gene Variants, Ki-67 and p53 in Pituitary Adenoma Pathophysiology
Source: Int J Mol Sci. 2025 Aug 5;26(15):7565. doi: 10.3390/ijms26157565 (PMC12347107; doi:10.3390/ijms26157565)
Supplement: Supplementary file 1 [file ijms-26-07565-s001.zip › ijms-3752176-supplementary.pdf]

SUPPLEMENTARY MATERIAR FOR

"The Role of FGFR4 (rs351855 and rs7708357) Gene Variants, Serum Levels, and Immunohistochemical Markers (Ki-67 and p53) in Association with Pituitary Adenoma"

Table S1. Binary logistic regression analysis of *FGFR4* rs351855 and rs7708357 in patients with PA and control groups

***FGFR4* (rs351855)**				
**Model**	**Genotype/Allele**	**OR (95% CI)**	***p*-value**	**AIC**
Codominant	GA *vs.* GG<br>AA *vs.* GG	1.124 (0.685-1.846)<br>0.974 (0.348-2.730)	0.643<br>0.961	385.665
Dominant	GA+AA *vs.* GG	1.106 (0.683-1.790)	0.682	383.741
Recessive	AA *vs.* GG+GA	0.918 (338-2.492)	0.867	382.880
Overdominant	GA *vs.* GG+AA	1.128 (0.697-1.824)	0.624	383.668
Additive	A	1.056 (0.711-1.567)	0.788	383.836
***FGFR4* (rs7708357)**				
**Model**	**Genotype/Allele**	**OR (95% CI)**	***p*-value**	**AIC**
Codominant	GA *vs.* GG<br>AA *vs.* GG	1.184 (0.709-1.975)<br>0.946 (0.424-2.110)	0.519<br>0.892	385.347
Dominant	GA+AA *vs.* GG	1.132 (0.694-1.844)	0.620	383.662
Recessive	AA *vs.* GG+GA	0.865 (0.407-1.838)	0.706	383.765
Overdominant	GA *vs.* GG+AA	1.198 (0.741-1.939)	0.461	383.366
Additive	A	1.034 (0.723-1.478)	0.855	383.875

OR: odds ratio; CI: confidence interval; AIC: Akaike information criteria; p-value: significance level (statistically significant when $p < 0.05$).

Table S2. Binary logistic regression analysis of *FGFR4* rs351855 and rs7708357 in female patients with PA and the control group

***FGFR4* (rs351855)**				
**Model**	**Genotype/Allele**	**OR (95% CI)**	***p*-value**	**AIC**
Codominant	GA *vs.* GG<br>AA *vs.* GG	1.401 (0.761-2.581)<br>0.852 (0.214-3.387)	0.279<br>0.820	256.367
Dominant	GA+AA *vs.* GG	1.332 (0.733-2.418)	0.347	254.905
Recessive	AA *vs.* GG+GA	0.716 (0.187-2.738)	0.625	255.546
Overdominant	GA *vs.* GG+AA	1.426 (0.788-2.581)	0.241	255.420
Additive	A	1.159 (0.710-1.893)	0.554	255.447
***FGFR4* (rs7708357)**				
**Model**	**Genotype/Allele**	**OR (95% CI)**	***p*-value**	**AIC**
Codominant	GA *vs.* GG<br>AA *vs.* GG	1.120 (0.597-2.103)<br>0.747 (0.265-2.107)	0.724<br>0.581	257.139
Dominant	GA+AA *vs.* GG	1.040 (0.568-1.905)	0.899	255.780
Recessive	AA *vs.* GG+GA	0.701 (0.264-1.860)	0.476	255.264
Overdominant	GA *vs.* GG+AA	1.194 (0.660-2.157)	0.558	255.453
Additive	A	0.943 (0.602-1.478)	0.799	255.731

OR: odds ratio; CI: confidence interval; AIC: Akaike information criteria; p-value: significance level (statistically significant when p < 0.05).

Table S3. Binary logistic regression analysis of *FGFR4* rs351855 and rs7708357 in male patients with PA and control groups

***FGFR4* (rs351855)**				
**Model**	**Genotype/Allele**	**OR (95% CI)**	***p*-value**	**AIC**
Codominant	GA *vs.* GG<br>AA *vs.* GG	0.722 (0.305-1.712)<br>1.083 (0.216-5.436)	0.460<br>0.923	130.381
Dominant	GA+AA *vs.* GG	0.765 (0.334-1.752)	0.526	128.618
Recessive	AA *vs.* GG+GA	1.273 (0.268-6.042)	0.762	128.929
Overdominant	GA *vs.* GG+AA	0.714 (0.310-1.644)	0.429	128.391
Additive	A	0.877 (0.448-1.718)	0.702	128.873
***FGFR4* (rs7708357)**				
**Model**	**Genotype/Allele**	**OR (95% CI)**	***p*-value**	**AIC**
Codominant	GA *vs.* GG<br>AA *vs.* GG	1.391 (0.572-3.386)<br>1.429 (0.388-5.264)	0.467<br>0.592	130.389
Dominant	GA+AA *vs.* GG	1.400 (0.608-3.223)	0.429	128.391
Recessive	AA *vs.* GG+GA	1.221 (0.357-4.179)	0.750	128.920
Overdominant	GA *vs.* GG+AA	1.287 (0.556-2.976)	0.555	128.673
Additive	A	1.247 (0.687-2.264)	0.469	128.496

OR: odds ratio; CI: confidence interval; AIC: Akaike information criteria; p-value: significance level (statistically significant when p < 0.05).

Table S4. Binary logistic regression analysis of *FGFR4* rs351855 and rs7708357 in the PA and control groups by PA tumor size

***FGFR4* (rs351855)**				
**Model**	**Genotype/Allele**	**OR (95% CI)**	***p*-value**	**AIC**
**Micro PA**				
Codominant	GA *vs.* GG<br>AA *vs.* GG	1.446 (0.702-2.976)<br>0.974 (0.200-4.755)	0.317<br>0.974	211.918
Dominant	GA+AA *vs.* GG	1.387 (0.684-2.814)	0.364	210.183
Recessive	AA *vs.* GG+GA	0.799 (0.173-3.694)	0.774	210.931
Overdominant	GA *vs.* GG+AA	1.450 (0.722-2.912)	0.296	209.919
Additive	A	1.201 (0.682-2.113)	0.526	210.619
**Macro PA**				
**Model**	**Genotype/Allele**	**OR (95% CI)**	***p*-value**	**AIC**
Codominant	GA *vs.* GG<br>AA *vs.* GG	0.964 (0.535-1.738)<br>0.974 (0.295-3.214)	0.902<br>0.966	290.706
Dominant	GA+AA *vs.* GG	0.965 (0.546-1.707)	0.903	288.706
Recessive	AA *vs.* GG+GA	0.992 (0.311-3.161)	0.989	288.721
Overdominant	GA *vs.* GG+AA	0.967 (0.545-1.713)	0.908	288.707
Additive	A	0.975 (0.611-1.577)	0.916	288.710
***FGFR4* (rs7708357)**				
**Model**	**Genotype/Allele**	**OR (95% CI)**	***p*-value**	**AIC**
**Micro PA**				

Codominant	GA *vs.* GG	1.095 (0.515-2.330)	0.813	212.670
	AA *vs.* GG	1.376 (0.483-3.917)	0.550	
Dominant	GA+AA *vs.* GG	1.157 (0.570-2.348)	0.687	210.854
Recessive	AA *vs.* GG+GA	1.312 (0.499-3.453)	0.582	210.726
Overdominant	GA *vs.* GG+AA	1.010 (0.503-2.028)	0.979	211.017
Additive	A	1.154 (0.700-1.905)	0.574	210.703
**Macro PA**				
**Model**	**Genotype/Allele**	**OR (95% CI)**	***p*-value**	**AIC**
Codominant	GA *vs.* GG	1.237 (0.678-2.257)	0.488	289.255
	AA *vs.* GG	0.688 (0.239-1.983)	0.489	
Dominant	GA+AA *vs.* GG	1.116 (0.625-1.993)	0.709	288.582
Recessive	AA *vs.* GG+GA	0.614 (0.225-1.679)	0.342	287.737
Overdominant	GA *vs.* GG+AA	1.330 (0.752-2.354)	0.327	287.760
Additive	A	0.961 (0.625-1.477)	0.856	288.688

OR: odds ratio; CI: confidence interval; AIC: Akaike information criteria; p-value: significance level (statistically significant when $p < 0.05$).

Table S5. Binary logistic regression analysis of *FGFR4* rs351855 and rs7708357 in the PA and control groups by PA invasiveness

***FGFR4* (rs351855)**				
**Model**	**Genotype/Allele**	**OR (95% CI)**	***p*-value**	**AIC**
**Non-invasive PA**				
Codominant	GA *vs.* GG	1.640 (0.838-3.209)	0.149	242.267
	AA *vs.* GG	1.290 (0.332-5.011)	0.713	
Dominant	GA+AA *vs.* GG	1.597 (0.828-3.078)	0.162	240.399
Recessive	AA *vs.* GG+GA	0.981 (0.268-3.590)	0.977	242.396
Overdominant	GA *vs.* GG+AA	1.585 (0.834-3.010)	0.160	240.397
Additive	A	1.349 (0.805-2.261)	0.255	241.111
**Invasive PA**				
**Model**	**Genotype/Allele**	**OR (95% CI)**	***p*-value**	**AIC**
Codominant	GA *vs.* GG	0.811 (0.433-1.520)	0.514	263.238
	AA *vs.* GG	0.783 (0.208-2.943)	0.717	
Dominant	GA+AA *vs.* GG	0.808 (0.440-1.482)	0.490	261.241
Recessive	AA *vs.* GG+GA	0.863 (0.237-3.147)	0.823	261.666
Overdominant	GA *vs.* GG+AA	0.833 (0.451-1.538)	0.559	261.374
Additive	A	0.844 (0.508-1.401)	0.512	261.282
***FGFR4* (rs7708357)**				
**Model**	**Genotype/Allele**	**OR (95% CI)**	***p*-value**	**AIC**
**Non-invasive PA**				
Codominant	GA *vs.* GG	1.546 (0.760-3.144)	0.229	242.286
	AA *vs.* GG	1.835 (0.698-4.824)	0.219	
Dominant	GA+AA *vs.* GG	1.609 (0.820-3.158)	0.167	240.418
Recessive	AA *vs.* GG+GA	1.436 (0.602-3.422)	0.414	241.759
Overdominant	GA *vs.* GG+AA	1.301 (0.689-2.459)	0.417	241.738
Additive	A	1.388 (0.878-2.195)	0.161	240.440
**Invasive PA**				
**Model**	**Genotype/Allele**	**OR (95% CI)**	***p*-value**	**AIC**

Codominant	GA *vs.* GG<br>AA *vs.* GG	0.966 (0.515-1.812)<br>0.413 (0.115-1.481)	0.915<br>0.175	261.424
Dominant	GA+AA *vs.* GG	0.845 (0.460-1.551)	0.587	261.422
Recessive	AA *vs.* GG+GA	0.420 (0.122-1.449)	0.170	259.436
Overdominant	GA *vs.* GG+AA	1.114 (0.607-2.044)	0.728	261.597
Additive	A	0.774 (0.484-1.238)	0.774	260.546

OR: odds ratio; CI: confidence interval; AIC: Akaike information criteria; p-value: significance level (statistically significant when $p < 0.05$).

Table S6. Binary logistic regression analysis of *FGFR4* rs351855 and rs7708357 in the PA and control groups by PA activity

FGFR4 **(rs351855)**				
**Model**	**Genotype/Allele**	**OR (95% CI)**	***p*-value**	**AIC**
**Non-active PA**				
Codominant	GA *vs.* GG<br>AA *vs.* GG	0.878 (0.433-1.780)<br>1.462 (0.431-4.950)	0.718<br>0.542	223.194
Dominant	GA+AA *vs.* GG	0.950 (0.485-1.861)	0.881	221.810
Recessive	AA *vs.* GG+GA	1.555 (0.480-5.035)	0.461	221.325
Overdominant	GA *vs.* GG+AA	0.832 (0.421-1.642)	0.595	221.548
Additive	A	1.053 (0.613-1.807)	0.852	221.798
**Active PA**				
**Model**	**Genotype/Allele**	**OR (95% CI)**	***p*-value**	**AIC**
Codominant	GA *vs.* GG<br>AA *vs.* GG	1.322 (0.728-2.400)<br>0.585 (0.124-2.761)	0.359<br>0.498	280.211
Dominant	GA+AA *vs.* GG	1.230 (0.685-2.211)	0.488	279.477
Recessive	AA *vs.* GG+GA	0.505 (0.111-2.303)	0.377	279.056
Overdominant	GA *vs.* GG+AA	1.391 (0.777-2.492)	0.267	278.722
Additive	A	1.058 (0.652-1.717)	0.820	279.909
FGFR4 **(rs7708357)**				
**Model**	**Genotype/Allele**	**OR (95% CI)**	***p*-value**	**AIC**
**Non-active PA**				
Codominant	GA *vs.* GG<br>AA *vs.* GG	1.710 (0.814-3.590)<br>1.323 (0.430-4.069)	0.157<br>0.625	221.765
Dominant	GA+AA *vs.* GG	1.625 (0.795-3.321)	0.183	219.995
Recessive	AA *vs.* GG+GA	0.972 (0.349-2.710)	0.957	221.829
Overdominant	GA *vs.* GG+AA	1.594 (0.810-3.136)	0.177	219.996
Additive	A	1.269 (0.776-2.075)	0.342	220.937
**Active PA**				
**Model**	**Genotype/Allele**	**OR (95% CI)**	***p*-value**	**AIC**
Codominant	GA *vs.* GG<br>AA *vs.* GG	0.931 (0.503-1.721)<br>0.764 (0.284-2.058)	0.818<br>0.595	281.665
Dominant	GA+AA *vs.* GG	0.894 (0.499-1.603)	0.707	279.820
Recessive	AA *vs.* GG+GA	0.792 (0.309-2.034)	0.629	279.718
Overdominant	GA *vs.* GG+AA	0.983 (0.548-1.763)	0.953	279.957
Additive	A	0.893 (0.578-1.380)	0.611	279.699

OR: odds ratio; CI: confidence interval; AIC: Akaike information criteria; p-value: significance level (statistically significant when $p < 0.05$).
